# Luteal and hypophyseal expression of the canine relaxin (RLN) system during pregnancy: Implications for luteotropic function

**DOI:** 10.1371/journal.pone.0191374

**Published:** 2018-01-24

**Authors:** Marta Nowak, Alois Boos, Mariusz P. Kowalewski

**Affiliations:** Institute of Veterinary Anatomy, Vetsuisse Faculty, University of Zurich, Zurich, Switzerland; Faculty of Animal Sciences and Food Engineering, University of São Paulo, BRAZIL

## Abstract

By acting through its receptors (RXFP1, RXFP2), relaxin (RLN) exerts species-specific effects during pregnancy; possible luteotropic effects through stimulation of prolactin (PRL) release have been suggested. In the domestic dog (*Canis lupus familiaris)* serum PRL increases in pregnant bitches shortly after RLN appears in the circulation, and a possible functional relationship between the RLN and the PRL systems in regulating progesterone secretion has been implied. Therefore, here (Study 1) the luteal expression and localization of the RLN system was investigated by immunohistochemistry using custom-made antibodies and semi-quantitative PCR, at selected time points during gestation: pre-implantation (d. 8–12), post-implantation (d. 18–25), mid-gestation (d. 35–40) and at normal and antigestagen-induced luteolysis. Further, (Study 2) hypophyseal expression of the RLN system and its spatial association with PRL was assessed. Luteal expression of *RLN*, but not of its receptors, was time-dependent: it increased significantly following implantation towards mid-gestation and decreased at prepartum. Antigestagen treatment resulted in downregulation of *RLN* and *RXFP2*. Whereas RLN was localized in steroidogenic cells, RXFP1 and RXFP2 also stained strongly in macrophages and vascular endothelial cells. The RLN system was detected in the canine adenohypophysis and was co-localized with PRL in hypophyseal lactotrophs. The intraluteal RLN seems to be involved in regulating the canine corpus luteum (CL) in a time-dependent manner. The presence of RLN family members in the adenohypophysis implies their possible involvement in regulating the availability of PRL and other pituitary hormones.

## Introduction

The peptide hormone relaxin (RLN) is involved in pregnancy-related remodeling of connective tissue [reviewed in [1, 2]]. Acting on the pubic ligament, pregnant uterus and uterine cervix, RLN participates in preparing the birth canal for parturition, thereby facilitating the course of labor. Although these effects of RLN are well-known and broadly described, several other actions of RLN are known to be species- and/or organ-specific. For instance, RLN was shown to be involved in decidualization and implantation in women [3, 4]. It was also found to act locally in an auto- and paracrine manner in the ovary by regulating follicular development in pigs [5] and humans [6] and, possibly, promoting ovulation in rats [7, 8]. In addition, due to the systemic distribution of its receptors, RXFP1 and RXFP2, the functions of RLN appear to spread beyond the reproductive tract [2].

RXFP1 and RXFP2 are members of the G-protein coupled receptor family (GPCRs). It is noteworthy that RLN is a cognate ligand for RXFP1, however it can bind and activate RXFP2 for which the main ligand is insulin-like peptide 3 (INSL3) [9]. The primary source of RLN is distinct for each species and can be localized in the corpus luteum (CL), placenta or uterus [1]. Moreover, besides its main origins, RLN is also synthesized in other reproductive organs, referred to as secondary sources, e.g., the ovary in mares [10] or the placenta in women [11]. These organs regulate their own function by local, auto- or paracrine secretion of RLN, without however contributing strongly to its circulating levels.

In contrast to other species like the pig, human or rat, in the domestic dog (*Canis lupus familiaris*), RLN is detectable only during gestation [12], which renders this hormone a useful endocrine diagnostic marker of pregnancy in this species. RLN becomes detectable in the blood around day 20–25 of pregnancy, peaks 2–3 weeks before whelping and stays elevated until term [13]. It originates predominantly from the placenta [14]. As revealed in our recent study [15], its major source appears to be cytotrophoblast. In the same study [15], RLN synthesis was confirmed in the uterus, i.e., endometrium and myometrium. Additionally, luteal expression of *RLN* mRNA and protein was previously reported [14, 16] in the dog. However, the CL seems to be a rather secondary source of RLN in this species, since ovariectomy performed during pregnancy does not affect circulating RLN [14].

The canine CL is an indispensible endocrine organ for pregnancy maintenance [17]. It is the only provider of progesterone (P4) in this species since the placenta is devoid of steroidogenic capacity [18, 19]. Progesterone secretion patterns are similar in pregnant and pseudopregnant dogs [18, 20]. The corresponding hormone levels vary widely individually, tending, however, to be higher during pregnancy [12] (reviewed in [20]). Interestingly, also taking into account increased plasma volume in pregnant bitches, similar concentrations of circulating P4 levels observed in pregnant and non-pregnant bitches [18], further suggest higher luteal steroidogenic activity during pregnancy. Consequently, it was hypothesized that during canine gestation, some factors, possibly placenta-derived, may act luteotropically by stimulating P4 synthesis [21]. Some authors suggested that increased circulating levels of prolactin (PRL) could indirectly originate in placental endocrine functions [17, 21, 22]. In fact, PRL is unequivocally needed for luteal maintenance during the second half of the CL lifespan in the bitch [23, 24]. This has been clearly shown in studies in which PRL secretion was suppressed by dopamine-agonist treatment, resulting in cessation of luteal function, and therefore termination of pregnancy [23, 24]. Furthermore, average serum PRL levels are typically higher in pregnant than in non-pregnant bitches [18]. However, because of high individual variations, and elevated PRL concentrations in overtly pseudopregnant bitches, clinical application of PRL as a reliable marker of gestation is precluded [24–26]. Consequently, the hypothesis was put forward that pregnancy-associated RLN might be responsible for elevated PRL levels during gestation [22]. This was supported by the observation that increased PRL secretion occurs concomitantly or shortly after the onset of placental RLN production, i.e., soon after implantation [21]. Observations from other species support this hypothesis. Thus, RLN stimulates secretion of PRL from the adenohypophysis in pigs [27] and rhesus monkeys [28] *in vivo*. Moreover, RLN stimulates PRL release from rat anterior pituitary cells *in vitro* [29]. However, the possible involvement of RLN in PRL secretion has never been established for the dog. Therefore, driven by the hypothesis that RLN might be indirectly involved in endocrine luteotropic regulation of canine CL by affecting hypophyseal PRL synthesis, here, for the first time we have investigated the expression and localization of the RLN system (i.e., RLN and its two receptors: RXFP1 and RXFP2) in the canine anterior pituitary and its co-localization in PRL-secreting cells. Moreover, as indicated by the foregoing data, there is still a lack of comprehensive information regarding time-dependent expression and localization of the RLN system in CL of pregnant dogs where RLN could potentially possess a local luteotropic function. For that reason, we investigated the spatio-temporal expression of the RLN system at both mRNA and protein levels in the canine CL during the course of pregnancy. The aim of our study was to investigate if the canine CL and hypophysis have the capability to produce and respond to RLN. Additionally, possible P4 receptor (PGR)-mediated effects were assessed in bitches treated with antigestagen (aglepristone) for premature induction of luteolysis/abortion.

## Materials and methods

### Animals and samples collection

(Study 1) Luteal tissue samples used in this study derived from our previous studies [30, 31]. All experiments were performed in accordance with guidelines of animal welfare legislation and were approved by the respective authorities, permits nos. II 25.3-19c20-15c GI 18/14 and VIG3-19c-20/15c GI 18,14 from Justus-Liebig University, Giessen, and permits nos. Ankara 2006/06 and 2008-25-124 from the Faculty of Veterinary Medicine, University of Ankara.

Briefly, healthy, crossbreed, sexually mature bitches (n = 29) were subjected to ovariohysterectomy (OHE). Immediately after surgery, tissues were placed in ice-cold PBS and transported to the laboratory. Using sterile scalpel blades, corpora lutea (CL) were dissected from surrounding ovarian tissue and processed either for mRNA or protein conservation as described below. CL were assigned to four study groups, representing the following stages of pregnancy: pre-implantation (days 8–12, n = 5), post-implantation (days 18–25, n = 5), mid-gestation (days 35–40, n = 6) and prepartum luteolysis (n = 3). Additionally, in order to terminate gestation, the antigestagen aglepristone (Alizine^®^, Virbac, Bad Oldesloe, Germany) at a dose of 10 mg/kg bw *s*.*c*.; 2x/24 h apart was administered in dogs at the mid-pregnancy stage (days 40–45 of pregnancy, n = 10). OHE was done 24h (n = 5) or 72 h (n = 5) after the second injection. The mean P4 concentrations were reported previously [30] and were as follows: 35.7±7.9ng/mL during pre-implantation, 29.7±13.2ng/mL at post-implantation, 13.3±8.6ng/mL at mid-gestation and 2.0±0.9ng/mL during the prepartum luteolysis. Following application of Alizine, P4 concentrations were as follows: 15.1±6.7ng/mL before the first application; 13.6±8.2ng/mL after the second treatment; 5.1±2.7ng/mL 24h later; 2.3±1.4ng/mL 48h later and 1.2±0.6ng/mL 72h later. No additional serum samples were available for the present study. The time point of ovulation was determined by regular measurements of peripheral serum P4 and vaginal cytology, and was considered to be the day when circulating P4 concentration for the first time exceeded 5ng/ml. Day 0 of pregnancy was then regarded as the day of mating that took place 2–3 days after ovulation. The pre-implantation group was determined by the presence of embryos flushed from the uteri. For the luteolytic group serum P4 was monitored at 6 h intervals starting from day 58 of pregnancy, and a decrease to at least 2-3ng/ml in three consecutive measurements was considered as the time of prepartum luteolysis. Canine endotheliochorial placenta starts to form immediately following implantation (days 17–18 after mating) and continues to develop during the first half of pregnancy reaching its maximal size around day 40 [32, 33]. From this time the utero-placental units are fully developed. Consequently, relating to the stages of placental development, the post-implantation and mid-gestation groups used in our study relate to time points in canine pregnancy characterized by the formation of, and the presence of the fully developed placentae, respectively.

Additionally, for (Study 2) the anterior part (adenohypophysis) of the pituitary gland was collected from three (n = 3) bitches (n = 2 in anestrus and n = 1 at diestrus). The tissue material for this part of the study was derived from other experiments performed at the University of Zurich under permit numbers 58/2013 and 171/2010 and was shared with us using the 3R (reduce, replace, refine) approach. The date of last heat had been recorded for each dog. The stage of the sexual cycle was additionally determined by macroscopic and histological evaluation of reproductive tracts. More accurate timing of the cycle stage was not possible. Purpose-bred beagle dogs from established breeding facility were used. On the day of euthanasia, the dogs were sedated with Acepromazine (0.1ml/10kg i.m., Prequillan^®^, Arovet). An intravenous catheter was placed and then dogs were euthanized by an intravenous pentobarbital (1ml/3kg Eutha^®^77, Veterinaria) overdose. Immediately after euthanasia, animals were transported to the laboratory. For removal of hypophyses, heads were separated from trunks, skulls were opened, hypophyses were identified and localized in the hypophyseal fossa (*Sella turcica*, Turkish saddle) of the sphenoid bone and removed. The entire procedure did not exceed 30 min.

Collected tissue samples were processed as described below for either paraffin embedding or RNA isolation. Thus, following surgery, tissue material was fixed for 24 h in 10% neutral phosphate-buffered formalin at +4°C. For the next seven days, samples were incubated at +4°C in phosphate-buffered saline (PBS), which was replaced daily. Afterwards, following dehydration in a graded ethanol series, tissue samples were embedded in paraffin-equivalent Histo-Comp (Vogel, Giessen, Germany) and subjected to immunohistochemistry (IHC). In order to extract total RNA, tissue materials were placed in RNAlater^®^ (Ambion Biotechnology GmbH, Wiesbaden, Germany) for 24h incubation at +4°C and subsequently frozen at -80°C until total RNA was isolated.

### Total RNA isolation, qualitative, conventional PCR, real time (TaqMan) qPCR and data evaluation

Total RNA from CL and adenohypophyses was extracted, using TRIzol^®^ reagent following the manufacturer’s instructions (Invitrogen, Carlsbad, CA, USA). A NanoDrop 2000C^®^ spectrophotometer (Thermo Fisher Scientific AG, Reinach, Switzerland) was used to assess the quality and quantity of isolated RNA. To eliminate contamination by genomic DNA, DNase treatment was performed using RQ1 RNase-free DNase (Promega, Duebendorf, Switzerland) according to the supplier’s instructions. Afterwards, using random hexamers as primers and as described before [34, 35], cDNA was prepared by the reverse transcription (RT) reaction. Reagents for RT were provided by Applied Biosystems by Thermo Fisher (Carlsbad, CA, USA). The final amount of cDNA of each sample corresponded to 100 ng of DNase-treated total RNA. Reactions were conducted in an Eppendorf Mastercycler^®^ (Vaudaux-Eppendorf AG, Basel, Switzerland). Based on published canine CDS sequences, primers (used in conventional, qualitative PCR and real time PCR) and TaqMan^®^ probes labeled with 6-carboxyfluorescein (6-FAM) and 6-carboxytetramethylrhodamine (TAMRA) were designed with Primer Express Software ver. 2.0 (Applied Biosystems) for three target genes: *RLN*, *RXFP1*, *RXFP2*, and a reference gene glyceraldehyde 3-phosphate dehydrogenase (*GAPDH*). A complete list of primers and TaqMan probes is provided in Table 1. Self-designed primers and TaqMan probes were purchased from Microsynth (Balgach, Switzerland). Canine-specific primers and probe mixtures for two reference genes, *B-ACTIN* (Prod. No. Cf03023880_g1) and *CYCLOPHILIN A* (Prod. No. Cf03986523-gH), were commercially available and purchased from Applied Biosystems. The efficiencies of the PCR assays were determined by the CT slope method assuring approximately 100% reaction efficiency. Selected, randomly chosen amplicons of all target genes were sent for commercial sequencing (Microsynth).

**Table 1:** List of primers used for real time (TaqMan) PCR.

Primers and TaqMan probes used for semi-quantitative RT-PCR			
Primer	Accession number	Primer sequence	Product length (bp)
*GAPDH*	AB028142	Forward: 5’-GCT GCC AAA TAT GAC GAC ATC A-3’	75
		Reverse: 5’-GTA GCC CAG GAT GCC TTT GAG-3’	
		TaqMan Probe: 5’-TCC CTC CGA TGC CTG CTT CAC TAC CTT-3’	
*RLN*	NM_001003132.1	Forward: 5’-TGC TAG GTG TCT GGC TGC TAC TAA-3’	94
		Reverse: 5’-CGG ACA TAA TCA CGA CCA CAT G-3’	
		TaqMan Probe: 5’-ACT TCC CAG AGA GAT CCC AGC CAC G-3’	
*RXFP1*	KY980750	Forward: 5’-GGC ACC AAT GGA GTG TGT TTC-3’	102
		Reverse: 5’-TGC CGC CAA GTT AAC ACC AA-3’	
		TaqMan Probe: 5’-TAC TGG AGC CCA GAT TTA TTC GGT GGC-3’	
*RXFP2*	NM_001005870.1	Forward: 5’-CCC TGT GGG AAT CTT ACC AAG T-3’	98
		Reverse: 5’-GTG TCA CCA CAG TTG TCC TCA TC-3’	
		TaqMan Probe: 5’-TTA CCC CGT GCT TTT CAC TGT GAT GGC-3’	
		Reverse: 5’-GGC AGT CTG CAC AAC AAA GG-3’	

Conventional, qualitative RT-PCR was performed using total RNA isolated from the anterior pituitary glands to verify expression of the RLN-system. The annealing temperature was set to 56°C. The hot start PCR reaction using the GeneAmp Gold RNA PCR Kit (Applied Biosystems, Foster City, CA, USA) was done as previously described [34]. Following this reaction, products were separated by electrophoresis using a 2% agarose gel and stained with ethidium bromide. Amplicons of all target genes were sent for commercial sequencing (Microsynth).

Regarding luteal expression of the RLN-system, an automated fluorometer ABI PRISM 7500 Sequence Detection System (Applied Biosystems) was used for semi-quantitative analysis of *RLN*-, *RXFP1*- and *RXFP2*-mRNA expression in each sample. Five μl of cDNA corresponding to 100 ng total RNA were used in a 25μl reaction mixture that consisted additionally of: 200 nM TaqMan Probe, 300 nM of each primer and 12.5μl Fast Start Universal Probe Master (ROX)^®^ (Roche Diagnostics, Mannheim, Germany). Each sample was run in duplicates in 96-well optical plates (Applied Biosystems). As negative controls, autoclaved water and the ‘minus-RT controls’ (i.e., samples treated with DNase but not subjected to RT) instead of cDNA were used. Amplification was done under the following conditions: initial denaturation for 10 min at 95°C, followed by 40 cycles for 15s at 95°C and 1 min at 60°C. Three reference genes, i.e., *GAPDH*, *B-ACTIN* and *CYCLOPHILIN A*, served for normalization of expression levels of the target genes, which were calculated using the comparative CT method (ΔΔCT method) in accordance with the ABI 7500 Fast Real-Time PCR System protocol provided by the manufacturer and as published previously [34, 35].

### Immunohistochemistry (IHC)

As previously reported [15], due to the fact that specific or cross-reacting antibodies were not commercially available, canine-specific polyclonal anti-RXFP1 and anti-RXFP2, custom-made antisera were generated (Eurogentec, Seraing, Belgium) in guinea pigs immunized against the following peptide sequences: RXFP1: C+AELDLGSNKIENLPP, amino acids 217–231 and C+ATEIRNQVKKEMILA, amino acids 532–546 of the canine RXFP1 sequence with GenBank accession number KY980750; RXFP2: C+QTSEVRNPIGREVAV, amino acids 605–619 and C+LLHKHRRKSIFKTKK, amino acids 688–702 of the canine RXFP2 sequence with GenBank accession number NM_001005870.1. Regarding detection of RLN, the anti-RLN, canine-specific polyclonal affinity purified anti-RLN custom-made antibody was generated in guinea pigs (Eurogentec) and targeted against the IPATDDKKLKAC peptide sequence, amino acids 23–34 of the canine RLN sequence with GenBank accession number NM_001003132.1. A cysteine (indicated as C+) amino acid was incorporated at the N- or C-terminal position of the peptide to target the coupling site on the carrier protein (Eurogentec). PRL in the canine adenohypophysis was detected using polyclonal rabbit anti-PRL antibody (ab186522, Abcam, Cambridge, UK) and macrophages in canine CL were detected using polyclonal rabbit anti-Major Histocompatibility Complex Class II (MHCII) antibody (orb101661, Biorbyt Limited, Cambridge, UK).

The immunohistochemistry procedure was carried out in accordance with our previously published protocol [34, 36]. In brief, tissue sections (2–3 μm in thickness) were mounted on SuperFrost microscope slides (Menzel-Glaeser, Braunschweig, Germany), dewaxed in xylene and rehydrated in a graded ethanol series. After washing for 5 min under tap water, antigens were retrieved in 10 mM citrate buffer pH 6.0, initially for 5 min at ambient temperature, then for 15 min in buffer heated in a microwave oven at 600W. Finally, slides were cooled down for 20 min. For RXFP1 localization in luteal samples, instead of heat-induced antigen retrieval, enzymatic digestion was done using 0.25% pepsin (Sigma-Aldrich Chemie GmbH, Buchs, Switzerland) in 10 mM HCl for 10 min at 37°C. Following antigen retrieval, slides were rinsed in tap water and placed in methanol with the addition of 0.3% hydrogen peroxide for quenching endogenous peroxidase activity. Subsequently, slides were washed for 5 min in IHC buffer/0.3% Triton X, pH 7.2–7.4 (0.8 mM Na_2_HPO_4_, 1.47 mM KH_2_PO_4_, 2.68 mM KCl, 137 mM NaCl) and nonspecific binding of antibodies was blocked by incubating with 10% goat serum for 20 min. Afterwards, samples were incubated overnight with primary antibodies at the following dilutions: a) for CL: 1:500 for anti-RLN antibody, 1:750 for anti-RXFP1 and 1:1000 for anti-RXFP2 canine-specific antisera, and 1:200 for anti-MHCII antibody; b) for hypophysis: 1:750 for anti-RLN antibody, 1:9000 for anti-PRL antibody, 1:1500 for anti-RXFP1 and 1:1000 for anti-RXFP2 canine-specific antisera. The specificity of IHC was evaluated using: 1) pre-immune guinea pig serum at the same dilution and protein concentration as the primary antiserum against RXFP1 and RXFP2, and 2) non-immune guinea pig IgG (for RLN) or rabbit IgG instead of the primary antibody against PRL and MHCII, as negative (isotype) controls. On the following day, after washing in IHC buffer/0.3% Triton X, slides were incubated for 30 min with biotinylated secondary antibodies at 1:100 dilution.

The goat anti-guinea pig secondary antibodies were used for detection of the RLN-system. For detection of PRL and MHCII goat anti-rabbit secondary antibodies were applied (both from Vector Laboratories, Burlingame, CA, USA). Afterwards, with washing steps in between, the streptavidin-peroxidase Vectastain ABC kit (Vector Laboratories) was added to enhance signals. After 30 min, and renewed washing in IHC buffer, peroxidase activity was detected using the Liquid DAB+ substrate kit (Dako, Basel, Switzerland). Following detection, hematoxylin served to counterstain the samples. After washing under tap water and dehydration in a graded ethanol series, slides were mounted in Histokit (Assistant, Osterode, Germany).

For localization of PRL and the RLN system in the adenohypophysis, 2 series (three and four slides, respectively) of consecutive sections of tissue were cut. Of these, the first two slides of each series were stained with hematoxylin and eosin (HE) and immunohistochemically for detection of PRL. The next slides were used in the IHC protocol for detection of RLN or RXFP1 and RXFP2.

### Statistical analysis

Normalization of data was done by logarithmic transformation. The geometric means (Xg) ± geometric standard deviation (SD) were calculated for the analysis of target gene expression. Next, in order to assess the effects of gestational stage on luteal RLN-system mRNA expression, a parametric one-way analysis of variance (ANOVA) was applied. In case of P<0.05 the Tukey-Kramer multiple comparisons post-test was performed and each pregnancy stage was compared with one another.

Regarding experimental groups treated with aglepristone, these were analyzed with reference to a non-treated mid-gestation group, which served as a non-treated control. Then, ANOVA was followed by Dunnett’s multiple comparisons test when P<0.05. Each of the treated groups (i.e., 24h and 72h after aglepristone treatment) was then compared with the mid-gestation group. All statistical analyses were done using the statistical software program GraphPad 3.06 (GraphPad Software, San Diego, CA, USA).

## Results

### (Study 1.1) Time-dependent expression of the RLN system in the canine CL throughout pregnancy

**Fig 1:**
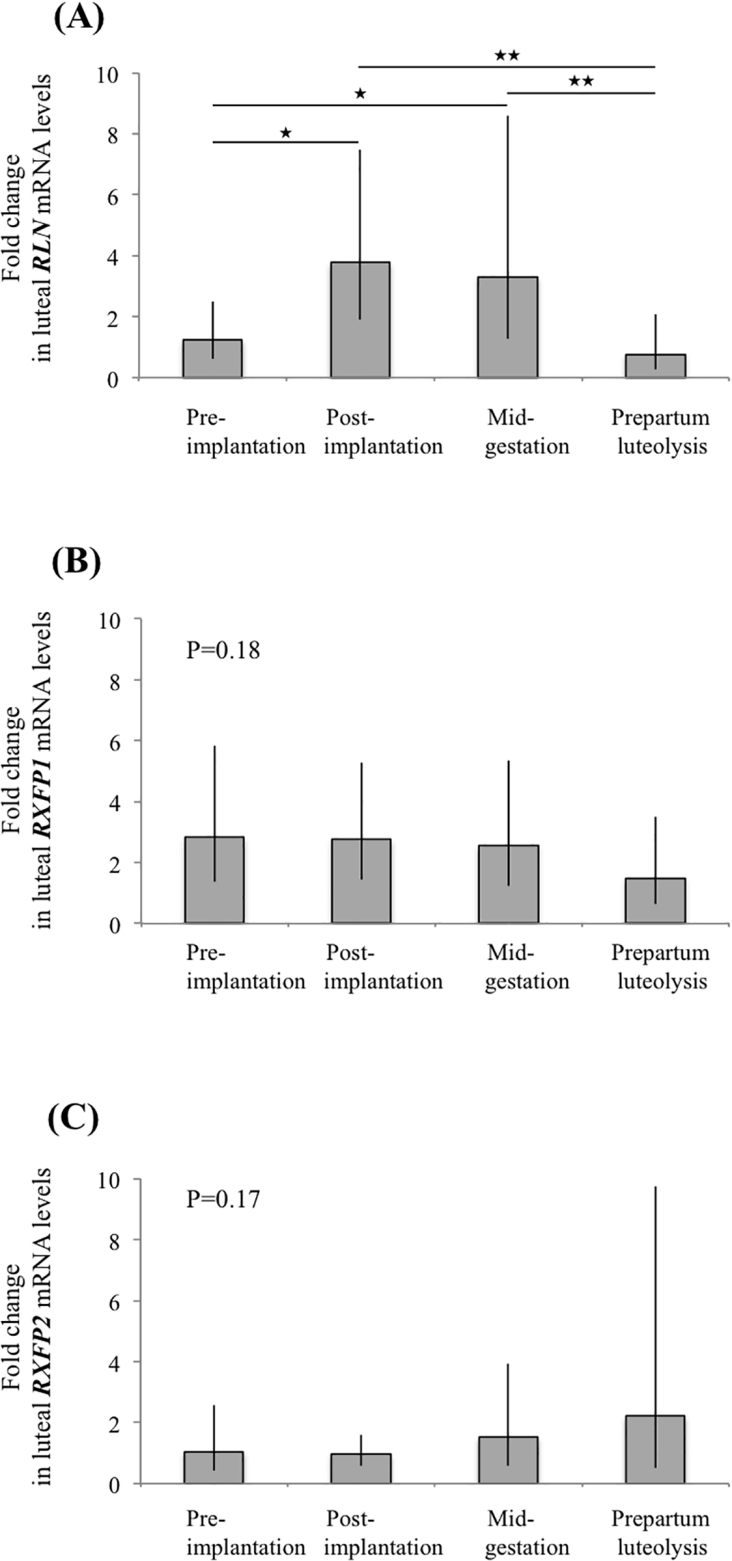
Expression of (A) *RLN*, (B) *RXFP1* and (C) *RXFP2* in canine CL at selected time points of pregnancy. Data are presented as geometric means Xg ± SD. (★) = P<0.01, (★★) = P<0.001.

The expression of transcripts encoding for RLN system members was detectable at all selected time points during pregnancy. *RLN* was significantly modulated in a time-dependent manner (P<0.0001) (Fig 1A). Its expression was upregulated from pre-implantation towards post-implantation (P<0.01), stayed elevated until mid-gestation and decreased significantly at prepartum luteolysis (P<0.001). The mRNA expression of both receptors did not change significantly over time (P>0.05); the mRNA levels of *RXFP2* varied widely individually and were below detection limits in two dogs, one from the pre-implantation stage and one from mid-gestation (Fig 1B and 1C).

### (Study 1.2) The effect of antigestagen-treatment on luteal expression of the RLN system

**Fig 2:**
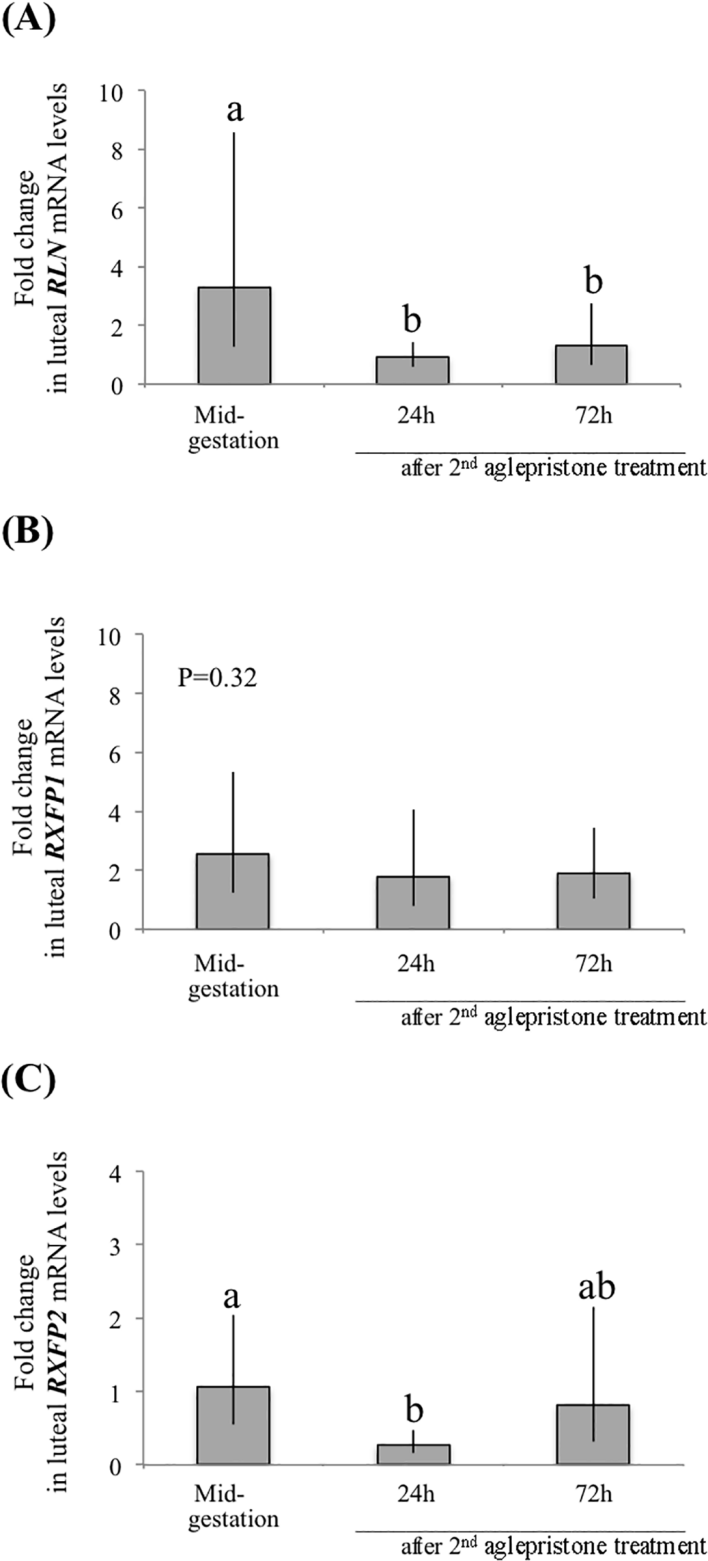
Antigestagen (aglepristone) effects on luteal expression of the RLN system (A) *RLN*, (B) *RXFP1* and (C) *RXFP2*), as determined by real time (TaqMan) PCR. Data are presented as geometric means Xg ± SD. Bars with different letters differ at P<0.01 compared to the mid-gestation group as a non-treated control.

The effects of antigestagen treatment on luteal expression of the RLN system were assessed in mid-pregnant dogs. As indicated elsewhere, samples from untreated mid-pregnant dogs were used as controls. The *RLN* mRNA levels were affected by antigestagen and already decreased significantly (P<0.01) 24h after the second treatment (Fig 2A) and remained low at 72h (P<0.01). As for RLN receptors, whereas the luteal expression of *RXFP1* was not affected by aglepristone (P = 0.32) (Fig 2B), the mRNA levels of *RXFP2* varied widely individually but decreased strongly 24h after the second treatment (P<0.01) (Fig 2C), remaining below the detection limits in two dogs. Similarly, at 72h after the second aglepristone application strong individual variations in *RXFP2* mRNA were observed that were not significantly different (P>0.05) (Fig 2C).

### (Study 1.3) Immunohistochemical detection of the RLN system in canine CL throughout pregnancy

**Fig 3:**
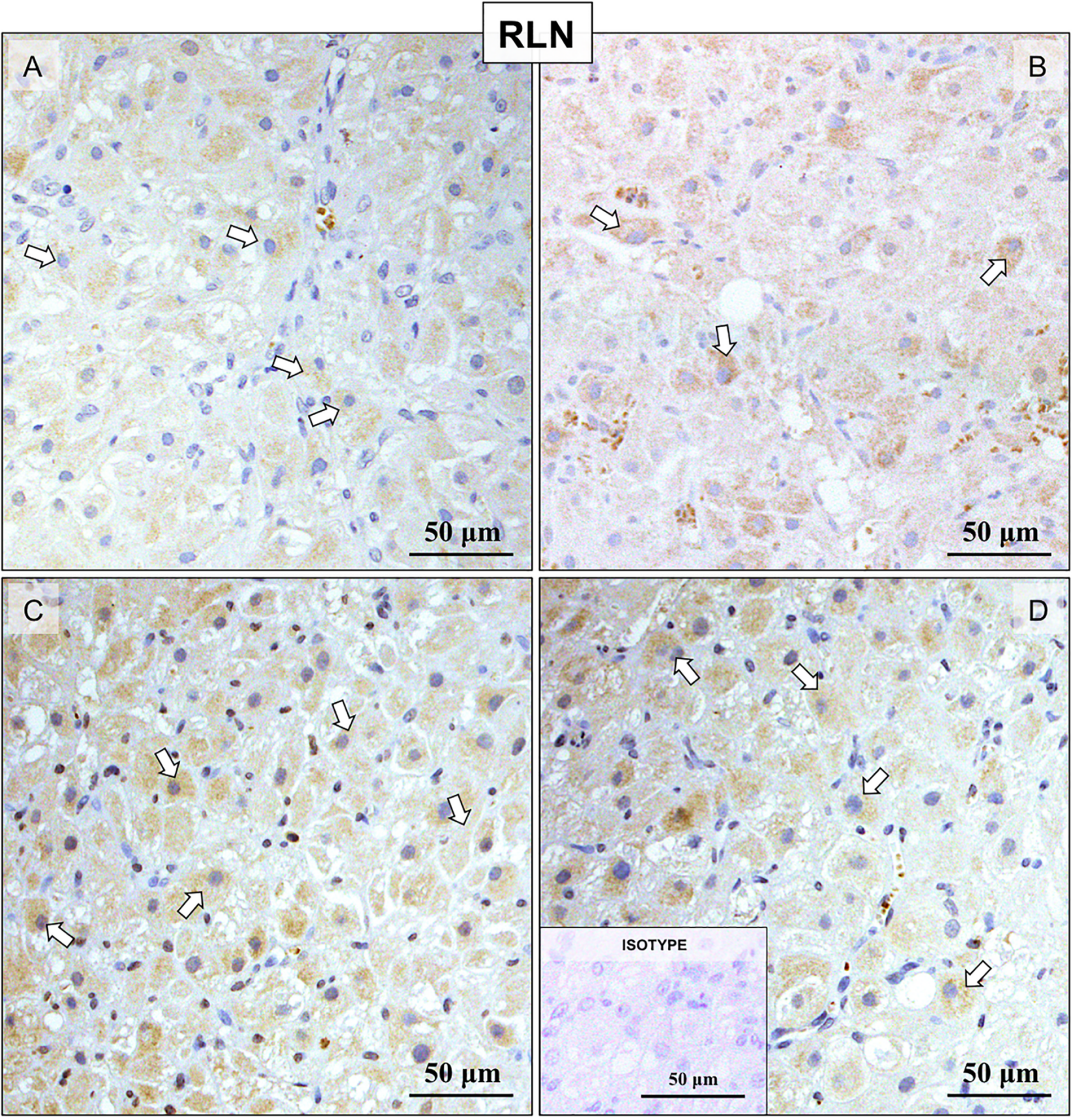
Representative microphotographs of immunohistochemical (IHC) localization of RLN in the canine CL at selected time points of pregnancy. Pre-implantation (A), post-implantation (B), mid-gestation (C) and at prepartum luteolysis (D). RLN is localized to the lutein cells (open arrows in (A–D)). There is no background staining in the isotype control (insert to (D)).

**Fig 4:**
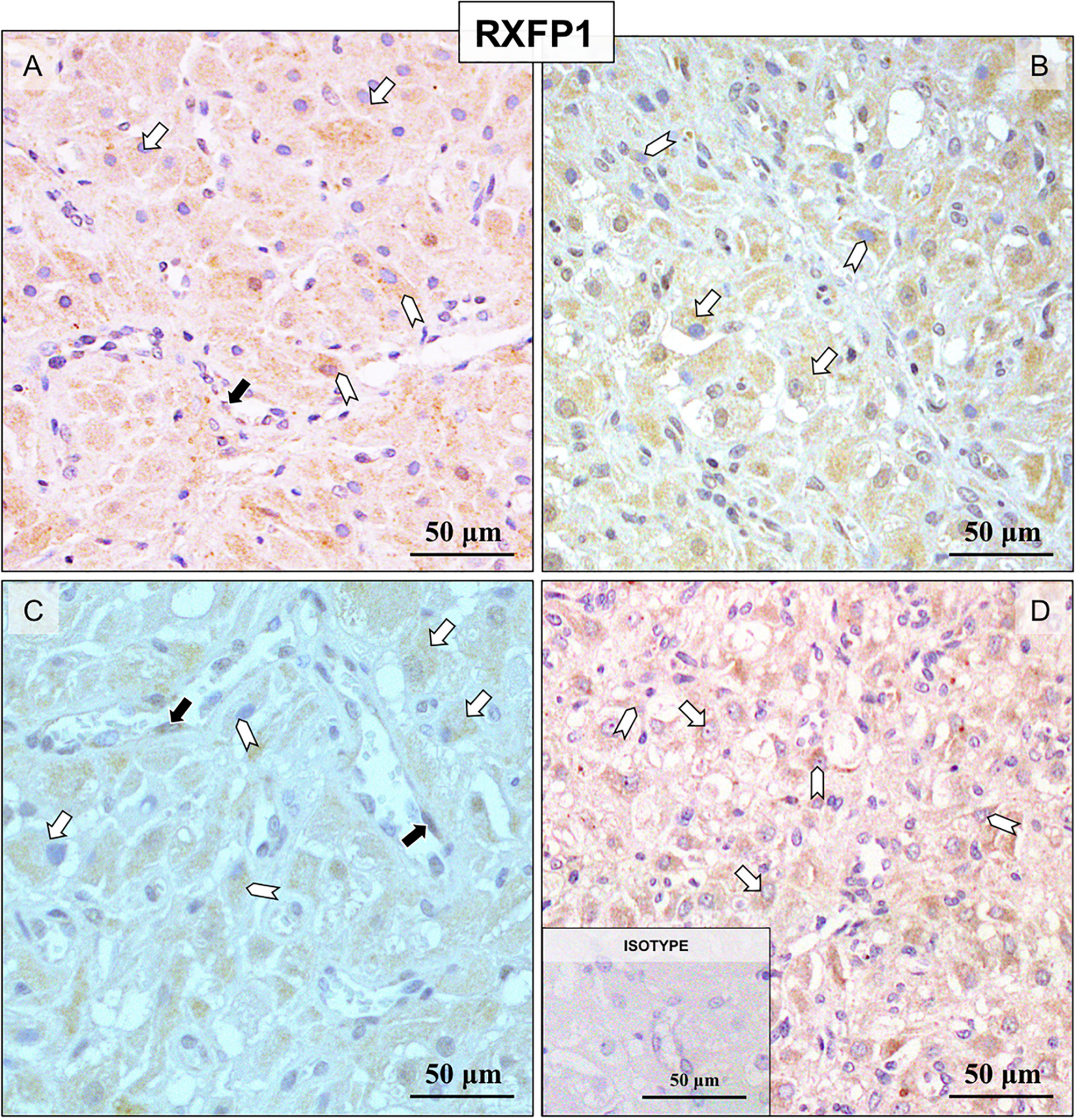
Representative microphotographs of immunohistochemical (IHC) localization of RXFP1 in the canine CL at selected time points of pregnancy. Pre-implantation (A), post-implantation (B), mid-gestation (C) and at prepartum luteolysis (D). RXFP1 is localized to the luteal cells (open arrows in (A-D)), endothelial cells (solid arrows in A and C) and macrophages (open arrowheads in (A-D)). There is no background staining in the isotype control (insert to (D)).

**Fig 5:**
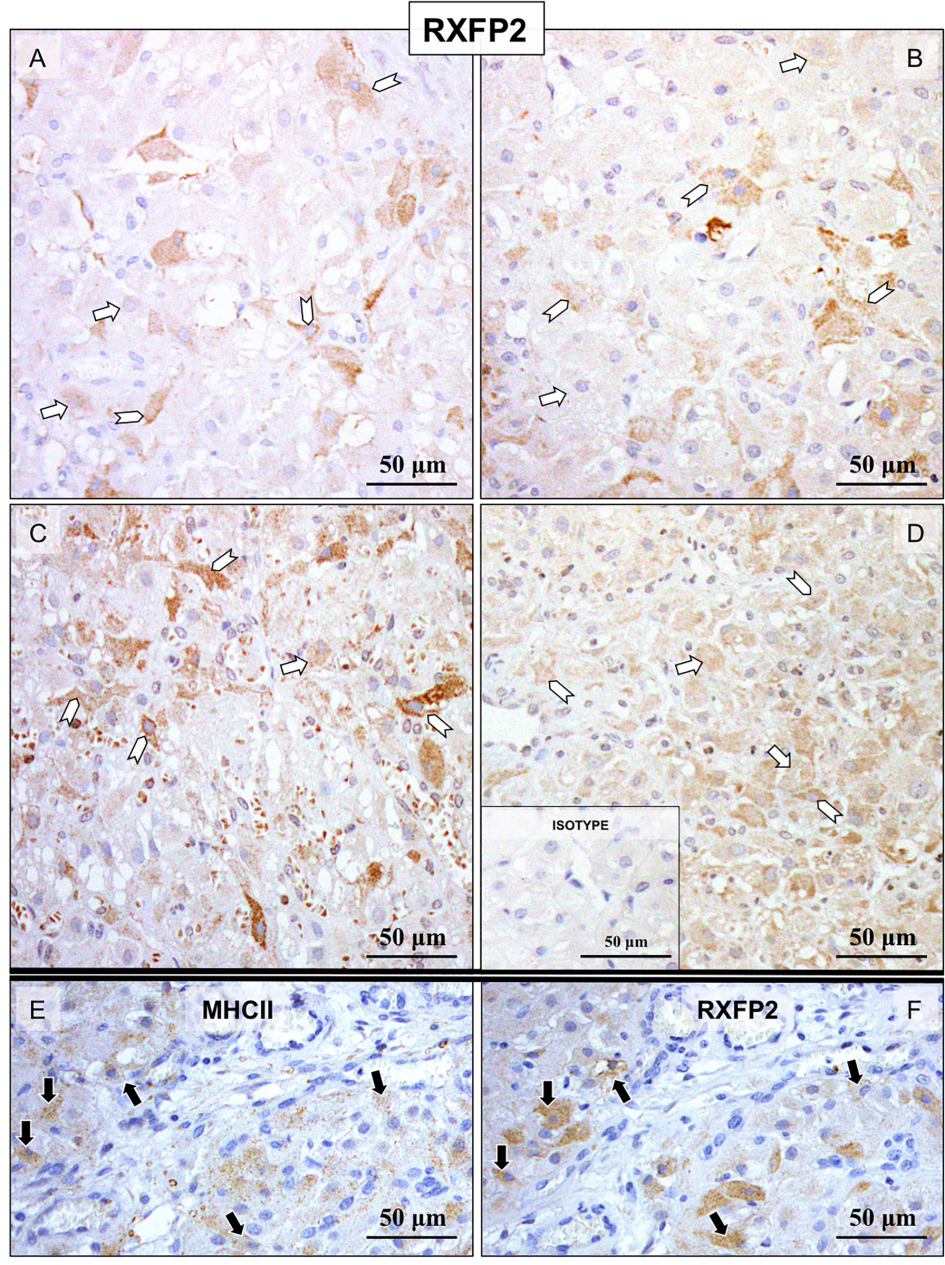
Representative microphotographs of immunohistochemical (IHC) localization of RXFP2 in the canine CL at selected time points of pregnancy. Pre-implantation (A), post-implantation (B), mid-gestation (C) and at prepartum luteolysis (D); consecutive sections were stained against MHCII (E) and RXFP2 (F). While only weak RXFP2 signals were found in luteal cells from pre-implantation until mid-gestation, during prepartum luteolysis clearly detectable staining is present in these cells (open arrows in A-D). The detection of MHCII was performed in order to more easily differentiate cell types within canine CL. Accordingly, RXFP2 was found in interstitial cells, mostly those localized close to blood vessels expressing MHCII that were identified as macrophages (black arrows in E and F, open arrowheads in A-D). There is no background staining in the isotype control (insert to (D)).

IHC was applied for detection of RLN, RXFP1 and RXFP2 localization at the protein level in canine CL at selected time points during pregnancy. Custom-made canine-specific antibody generated against RLN strongly stained luteal cells (Fig 3A–3D). Although large individual variations were observed, the signals appeared stronger at mid-gestation (Fig 3C) and prepartum luteolysis (Fig 3D). Other luteal compartments, e.g., stroma and vascular components, stained weakly. The immunolocalization of RXFP1 consisted mainly of luteal cells (Fig 4A–4D), however, the signal intensity appeared weaker at prepartum luteolysis (Fig 4D). Additionally, RXFP1 was found in endothelial cells (indicated in Fig 4A and 4C) and luteal macrophages (Fig 4A–4D). On the other hand, RXFP2 was detected primarily in interstitial cells (Fig 5A–5D), especially those localized in close proximity to blood vessels, throughout all stages of pregnancy. Detection of MHCII, a marker of antigen-presenting cells, shown recently to be predominantly localized in macrophages localized close to luteal blood vessels in canine CL [37], was performed in order to more easily differentiate cell types within canine CL (Fig 5E). Accordingly, the interstitial cells staining strongly for RXFP2 were identified as luteal macrophages (Fig 5E and 5F). In addition, whereas only weak signals were found in luteal cells from pre-implantation until mid-gestation, during prepartum luteolysis these cells clearly stained for RXFP2 (Fig 5A–5D). Occasionally, for all antibodies some background signals were observed in erythrocytes.

### (Study 2) Expression and localization of PRL and the RLN system in canine adenohypophysis

**Fig 6:**
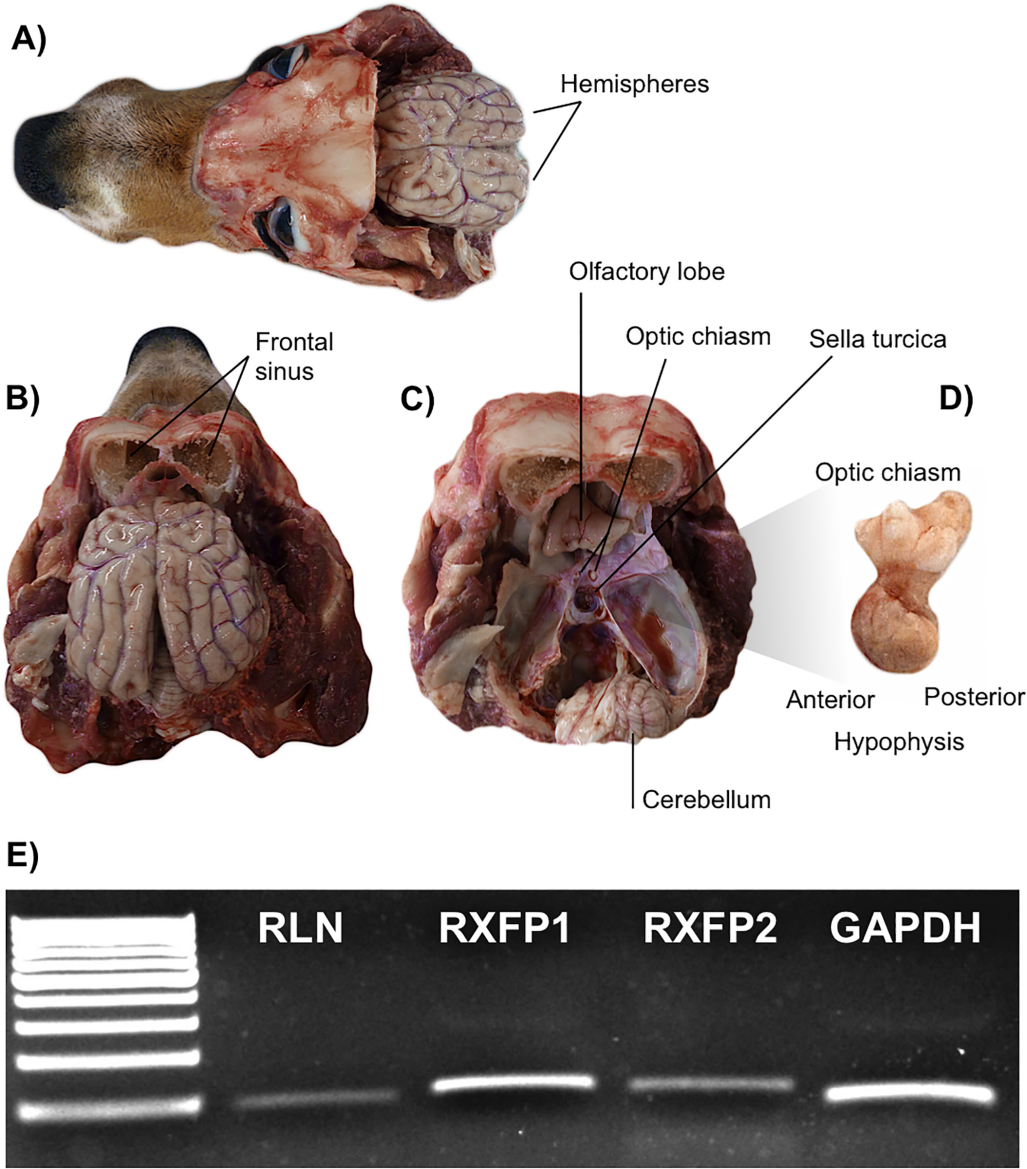
Isolation of hypophysis and adehohypophyseal expression of RLN-system. Representative photographs of opened skull of a dog before (A, dorsal view and B, dorsocaudal view), and after (C, dorsocaudal view) removal of the brain including the hypophysis (D). The hypophysis was localized in the saddle-shaped depression of the sphenoid bone, so-called Turkish saddle (*Sella turcica*). Proximally, directly above the pituitary gland, the optic chiasm can be localized. Parts of the optic chiasm are attached to the isolated hypophysis (D). E) Expression of *RLN*, *RXFP1*, *RXFP2* and *GAPDH* in canine adenohypophysis by conventional, qualitative PCR. The mRNA of all RLN system members was detectable, with *RLN* and *RXFP2* signals appearing weaker than those for *RXFP1*.

**Fig 7:**
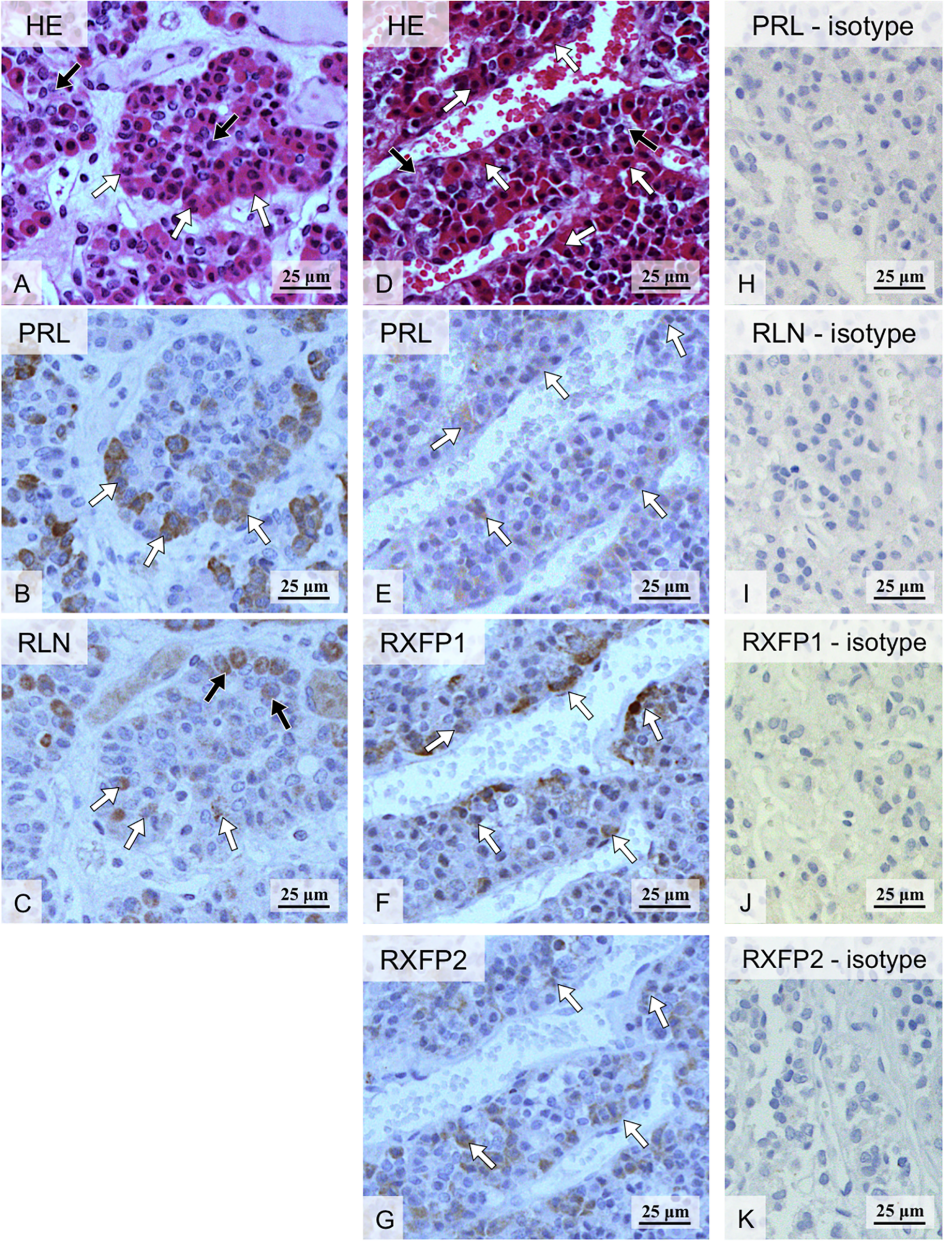
Hematoxylin-eosin (HE) staining (A, D) and immunolocalization of PRL (B, E) and RLN system (C, F, G) in canine adenohypophysis (two series of consecutive sections are presented: A-C and D-G). HE staining was performed in order to distinguish basophilic (solid arrows in A and D) and acidophilic hypophyseal cells (open arrows in A and D). The latter include PRL-secreting cells, so-called lactotrophs, identified additionally by IHC with anti-PRL antibody (open arrows in B and E). RLN signals were observed in lactotrophs (open arrows in C) and in other hypophyseal cells, which remained negative for PRL (solid arrows in C). RXFP1 and RXFP2 stained extensively in hypophyseal cells with signals appearing more prominent for RXFP1 than RXFP2 (open arrows in F and G, respectively). There is no background staining in the isotype controls (H, I, J, K).

Following the removal of hypophyses from the skull, anterior and posterior parts of the organ (adenohypophysis and neurohypophysis) were identified and separated (Fig 6A–6D). Expression of the RLN system was confirmed in the canine adenohypophysis by qualitative, conventional PCR (Fig 6E), although the intensity of *RLN* and *RXFP2* signals appeared weaker than for *RXFP1*. Their specificity was, however, confirmed by sequencing. In order to investigate the hypophyseal localization of the RLN system and PRL at the protein level, IHC was applied. The HE staining allowed us to distinguish between the acidophilic and basophilic compartments of the gland. Consequently, PRL staining was found in some acidophilic cells in consecutively stained tissue sections (Fig 7A, 7B, 7D and 7E). This localization pattern was further observed for RLN, which was co-localized with PRL in the same cellular components (Fig 7C). However, additional RLN signals were also detected in other hypophyseal cells, which remained negative for PRL. RLN receptors were ubiquitously distributed in the canine adenohypophysis, in both acidophilic and basophilic compartments, with signals appearing more prominent for RXFP1 than RXFP2 (Fig 7F and 7G). There was no background staining observed in negative/isotype controls (Fig 7H–7K).

## Discussion

With this study, we aimed to evaluate the presumptive luteotropic function of RLN in the canine species. Therefore, the basic capability of canine CL to produce and respond to RLN was assessed at selected time points of pregnancy from the pre-implantation stage until prepartum luteolysis. Moreover, here for the first time, the hypophyseal cellular expression and localization of the RLN system in the dog is described, providing further insights into possible RLN-mediated effects on PRL secretion in the dog.

In Study 1, luteal development was associated with increasing expression of RLN, but not of its receptors. The highest *RLN* mRNA levels were observed in fully functional CL during post-implantation and at mid-gestation, apparently coinciding with the maximal steroidogenic capabilities of the CL. Thus, at least a supporting role of locally-produced RLN in regulating canine CL is suggested. The acute suppression of luteal RLN expression during normal and antigestagen-induced luteolysis implies a functional positive feedback loop between RLN and P4. Possible interactions with downstream responders of P4, or other P4-mediated regulatory pathways, could be additionally involved. The possible luteal function of RLN appears to depend, however, more strongly on its own expression, than on the availability of its receptors, i.e., RXFP1 and RXFP2, which seem to be constantly present during most of the luteal life span.

A wide spectrum of RLN targets within the canine CL was observed in this study, implying that the functionality of RLN varies among luteal components. The strong expression of the RLN system, especially RLN and RXFP1, in steroidogenic cells indicates possible direct effects of RLN on their function. Moreover, RLN seems to be involved in regulating the function of luteal vessels, acting particularly on endothelial cells, in which predominantly RXFP1 was clearly represented. Knowing that vasodilatory properties of RLN [38, 39] are related to stimulation of nitric oxide (NO) production [reviewed in [40]], it seems plausible that RLN could also participate in regulation of the luteal microcirculation in the dog. Furthermore, RLN was shown to increase expression of vascular endothelial growth factor (VEGF) in human endometrial cells [3]. Considering that *VEGF* is expressed in a time-dependent manner in canine CL and that its expression increases post-implantation [41], a possible interaction between these two systems (i.e., the RLN and VEGF systems) could also apply to the canine species. Nevertheless, the characteristics of this interplay need further clarification.

An interesting finding from our study was the detection of RLN receptors, mainly RXFP2, in luteal macrophages. This observation implies possible involvement of RLN in regulating luteal immune cells. In accordance with this, by supporting vascular formation, luteal macrophages were shown to play a role in coordinating the development of human CL [42]. In another *in vitro* study, RLN mediated release of cytokines from human decidual macrophages [43]. Taking these facts into account, our results suggest that RLN-mediated effects on luteal macrophages could possibly be exerted through interaction with RXFP2. It should be noted, however, that INSL3, formerly known as relaxin-like factor (RLF), is a cognate agonist for RXFP2, which is a minor receptor for RLN. Thus, the possible involvement of INSL3 in regulation of luteal macrophages cannot be excluded and indicates a direction for future studies.

Administration of a P4 receptor (PGR) antagonist, aglepristone, to dogs at mid-gestation results in activation of the placental prostaglandin system, premature luteolysis and finally in a rapid decline of P4 [44]. Apart from *RLN*, the expression of one of its receptors, *RXFP2*, was also strongly affected by antigestagen treatment. The involvement of P4 signaling in this process appears plausible. This would resemble the situation observed *in vitro* with human granulosa cells, which when treated with a PGR antagonist responded with a dramatic decline in secretion of estrogens, P4 and RLN [45]. It is noteworthy, however, that although PGR-mediated effects appear to affect luteal and placental [15] RLN expression in dogs, they do not seem to interfere directly with circulating RLN [46, 47] and, thus, to be relevant for the overall supply of RLN.

Next, following observations made in other species, i.e., pigs and monkeys, in which positive, RLN-mediated effects on hypophyseal PRL secretion were observed [27, 28], we were prompted in Study 2 to investigate the presence and spatial association of the RLN system and PRL in the canine pituitary gland. This allowed us to address the hypothesis of potential indirect luteotropic function of RLN by stimulating hypophyseal PRL secretion in dogs.

It needs to be emphasized that the tissue material (i.e., canine pituitary glands) used in our study was not obtained with respect to a particular reproductive stage since the dogs were not euthanized for the purpose of this study. Nevertheless, we were able to verify the presence of the RLN system in adenohypophyses from dogs, both at anestrus and diestrus. The presence of its respective transcripts and proteins was clearly confirmed. Finally, co-localization with PRL-expressing cells was established. Based on the available literature [48], PRL is present in the serum of anestric dogs. Therefore, we expected to detect PRL in pituitary cells referred to as lactotrophs in the tissues collected during anestrus. For better differentiation of particular cellular components, the first consecutive sections stained for target proteins were assessed by HE staining. With regards to the RLN system, the signals for RXFP1 appeared stronger than for RXFP2, at both the mRNA and protein levels. Both receptors were found widely distributed in both cell types, i.e., acidophilic as well as basophilic hypophyseal cells. They clearly stained in lactotrophs, which were identified immunohistochemically with anti-PRL antibody. This strongly indicates that RLN, interacting with its receptors, could affect the function and secretory activity of the canine adenohypophysis. Notably, in addition to the above-cited *in vivo* studies performed in pigs and rhesus monkeys [27, 28], Sortino et al. [29] observed an increase in PRL release from rat anterior pituitary cells *in vitro* following stimulation with RLN, implying a central role of RLN in modulating PRL secretion in these species. That response occurred due to RLN-induced accumulation of cyclic adenosine monophosphate (cAMP) [49, 50]. Interestingly, the RLN effect on cAMP can be abolished by dopamine [49], a potent PRL inhibitor. In this context, it appears noteworthy that dopamine-agonists (e.g., cabergoline, bromocriptine) are routinely used in veterinary practice to decrease PRL levels and interrupt lactation in overtly pseudopregnant bitches.

Cumulatively, based on the presented results herein, the involvement of RLN in regulating the canine adenohypophysis, and possibly its PRL secretion, is strongly implied. Regrettably, owing to the lack of temporal accuracy in tissue collections, cycle and/or pregnancy stage-related effects could not be assessed and remain open for future studies. Moreover, since our study only provided hints for possible relationships among adenohypophyseal hormones, the effects of RLN on secretion of other pituitary hormones in the dog also remain to be elucidated.

In conclusion, by revealing the spatial and temporal expression of the RLN system in the CL of pregnant dogs and localizing it in the anterior pituitary gland, herein new insights are provided into canine reproductive physiology.

Although luteal RLN does not seem to contribute significantly to overall circulating levels of this hormone during pregnancy, our data suggest that by acting in an autocrine- and paracrine manner, RLN could possibly be directly involved in support of luteal steroidogenic function in the dog. The endocrine route could involve the interaction of placental RLN with its luteal receptors. Additionally, by interacting with endothelial cells and macrophages, RLN could potentially be involved in regulation of the microvascular system in canine CL.

**Fig 8:**
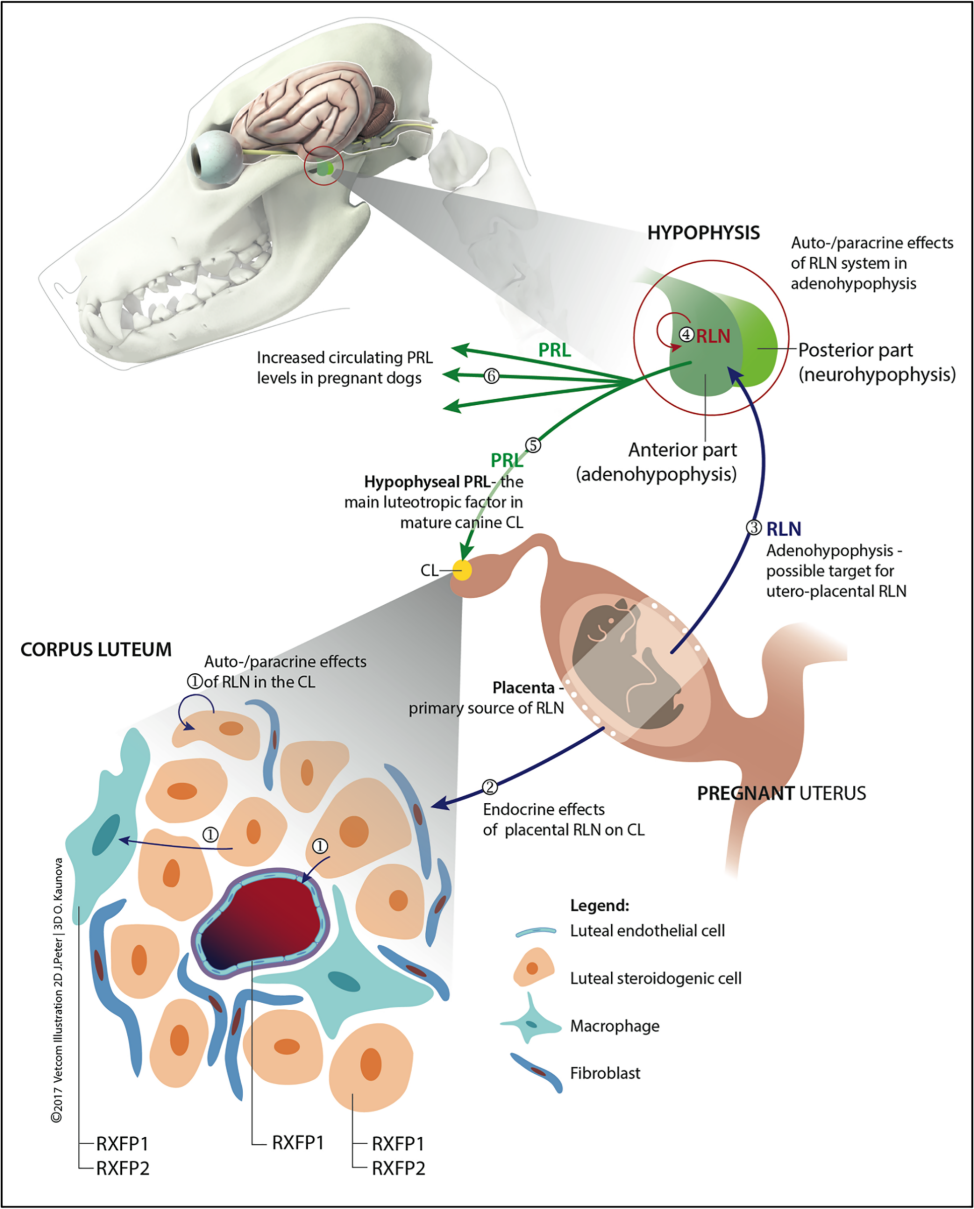
A schematic model depicting proposed aspects of autocrine, paracrine and endocrine functions of RLN in canine reproductive tissues, emphasizing its role as a possible luteotropic factor. (1) Local, luteal RLN acts in an auto/paracrine manner on steroidogenic, endothelial and immune cells. (2) Circulating RLN of placental origin acting as an endocrine factor. (3) Adenohypophysis, as a target of placental RLN during pregnancy. Additionally, locally produced hypophyseal RLN may exert auto/paracrine effects on pituitary cells (4); possible interactions of RLN with production and secretion of other hypophyseal hormones, including PRL are suggested. (5) PRL originating from the hypophysis is the main luteotropic hormone for mature canine CL [25]. Increased PRL levels observed following emergence of RLN (6) in circulation imply an indirect luteotropic function of RLN.

A schematic representation of the distribution pattern of RLN system in canine reproductive tissues and its possible interactions is shown in Fig 8.

The interplay between luteal RLN and P4 certainly deserves further attention since natural or induced withdrawal of P4 during luteolysis had a negative feedback on luteal RLN expression. The indirect effects of RLN on luteal function may rely on stimulation of PRL secretion from the adenohypophysis (Fig 8). The evident presence of RXFP1 and RXFP2 proves that the adenohypophysis is an important target for RLN. It appears especially plausible for lactotrophs since the PRL serum level increases significantly after RLN rises in the blood of a pregnant bitch (Fig 8).

Although a direct connection between RLN and PRL remains to be convincingly demonstrated, here we provide a novel aspect of RLN action with respect to the canine adenohypophysis.
